# Standard values of the upper body posture and postural control: a study protocol

**DOI:** 10.1186/s12995-016-0122-9

**Published:** 2016-07-16

**Authors:** Daniela Ohlendorf, Christoph Mickel, Natalie Filmann, Eileen M. Wanke, David A. Groneberg

**Affiliations:** Institute of Occupational Medicine, Social Medicine and Environmental Medicine, Goethe-University Frankfurt/Main, Theodor-Stern-Kai 7, Building 9A, Frankfurt/Main, 60590 Germany; Institute of Sport Sciences, Goethe-University Frankfurt/Main, Ginnheimer Landstraße 39, Frankfurt/Main, 60487 Germany; Institute of Biostatistics and Mathematical Modeling, Goethe-University, Frankfurt/Main, Theodor-Stern-Kai 7, Building 11, Frankfurt/Main, 60590 Germany

**Keywords:** Study protocol, Upper body posture, Postural control, Standard value, Confidence interval

## Abstract

**Background:**

Decisions on orthopedic interventions on upper body posture and its control have usually resulted from comparisons with the healthy state. Therefore, practitioners as well as scientists in human movement science or orthopedics need access to such kind of data which are patient-centered and well measured. Until now, these data have been missing concerning upper body posture as well as postural control and their control. That is why the aim of the current project is to measure these data with healthy participants across the lifespan.

**Results:**

For standard value determination tolerance range and confidence intervals will be calculated. In addition, Pearson- or Spearman-Rank correlations will be used as well as two-sample-t-tests or Mann-Whitney-U-tests for specific group differences. All tests will be two-sided with the level of significance of 5 %.

**Discussion:**

This project aims at improving classifications in adaptations of upper body posture and postural control. Measured standard values have not been determined before to this extent. Therefore, interventional effects may become better quantifiable and justiciable.

## Background

Postural control has to be regarded as a complex feedback-dependent system using various sensory inputs from visual, vestibular and somatosensory receptors [1–3]. Depending on what perception is being considered most important in any given task, the sensory information is rated and tested against each other. A common assumption is that the postural control system uses internal models of the body’s configuration and its dynamics, which are controlled by the central nervous system (= CNS). This control mechanism has to be highly adaptable at all times to be able to adjust to changing postural challenges. Those challenges, themselves, are highly variable, as they include short-term (e.g. surface changes) and long-term adaptations (e.g. physiological changes across the life span) [4–9].

Neuromuscular activity controls the position of all body segments in consideration of the external forces to produce a functional equilibrium. As all biological systems always try to minimize energetic costs for a given task, an optimal (in this sense) upright posture in humans is present if body weight is evenly distributed on both feet, as in this case the centre of mass (COM) is only vertically displaced from the centre of pressure (COP). Therefore, this body position may be maintained with the least possible muscular activity, as only weight load has to be supported while torques are minimized. Furthermore, the skeleton is minimally stressed [3, 10–12]. However, bipedal upright posture is an ‘unstable’ equilibrium, as has been shown by numerous studies using COP movement in static postural control: Even healthy individuals exhibit body sway. Further, the fluctuations are altered (mostly increased in spatial terms) if sensory perception is negatively influenced. Moreover, body constitution [13–15], age [5, 16], sex [16, 17] and maybe many more factors influence COP movement.

Measurement of postural control via COP tracking is an outcome measure analysis which is absolutely necessary and vital, but it is not able to give distinct information about alterations in upper body posture. It has repeatedly been shown that humans compensate for vertebral asymmetries which in case of a successful compensation cannot be detected via COP measurements anymore [18]. As wrong habitual posture may lead to adaptations/compensations of the trunk, the same applies vice versa. In consideration of the increasing number of back pain patients especially in industrialized nations, it is necessary to improve the quality of therapeutic and preventive interventions.

The decision which type of intervention is used for treatment is highly dependent on the experience of treating physician, physiotherapist, etc. and/or the patient him-/herself. Therefore, we are of the opinion that standard values of healthy persons of all age groups (>20 years) may and should help to improve the quality of current interventions.

From a scientific point of view, only if there are standard values guaranteeing objective classifications reliable and valid evaluations of adaptations can be made.

In the context of upper body posture and postural control, there has not been any documentation about time-synchronized measurements which could be used as standard values. If establishing standard values is the aim of any project, participant selection is crucial. In our case, participants will be from different social strata, and hence from different professions, while age and gender shall be distributed equally. Furthermore, a questionnaire evaluating risk factors, as for example, obesity, smoking, sedentary life style is used in search for further associations/correlations.

### Aims

In this project non-invasive measurement techniques shall be used to determine upper body posture synchronously to static postural control. Upper body posture will be analysed through videorasterstereography, which has been used for this purpose before [19–24]. Further, postural control data will be collected using posturography [25–28]. A time-synchronized measurement of upper body posture through three-dimensional back scan and posturography has never been done before, but is expected to offer new insights to their interdependence. Recent papers have proven the need for standard values in this setting [29–31]. Therefore, the main aim of this study project is to register a wide ranging database of 1000 healthy subjects to record upper body posture and postural control values. These data enable a general description of the upper body posture and the postural control during habitual standing by defining standard values and confidence intervals using all evaluation parameters.

As secondary aim besides the collection of personal data like age, height, weight (BMI) or gender additional information concerning sport related activities or rather doing sport, smoking behavior or the livelihood and duration of backache and other common complaints are collected by a questionnaire. Having these additional data the calculation of correlations and regressions, respectively, can be conducted.

Therefore, the following parameters will be analyzed in terms of age, gender, social strata and profession:

- Determination of a general range of tolerance and confidence intervals for upper body posture and postural control.
- Determination of range of tolerance and confidence intervals for upper body posture and postural control depending on age.
- Determination of range of tolerance and confidence intervals for upper body posture and postural control depending on gender.
- Correlations between height, weight or body-mass-index and upper body posture and postural control.
- Correlations between hours of work and upper body posture and postural control.
- Correlations between work activities (basically sitting, standing or a mixture of both) and upper body posture and postural control.
- Correlations between hours of work and work activities (basically sitting, standing or a mixture of both).
- Correlations between physical activity, age and upper body posture and postural control.

## Methods

### Study population

In the course of this project 1000 participants >20 years will be tested, whereby age and gender will be divided equally. Therefore, each age group (20–30, 31–40, 41–50, 51–60, >60 years) will consist of a group of 200 persons, from whom 100 will be female. Body height and weight will be measured, while foot size, laterality and existing physical complaints (migraine headache, rheumatism, joint aches, back pain and the prevalence, smoking habits, wearing orthopedic insoles) will be assessed. Further, occupational activity, e.g. type of professional practice (mainly seated or standing) or hours of daily physical activity, hours of work, years working this job, physical activity and playing an instrument will be asked for.

Exclusion criteria are any kind of previous treatment, such as surgery or accidents, of the musculoskeletal system as well as the temporomandibular system should date back at least 2 years.

This study was approved by the ethics board for research involving human subjects of the Goethe University (219/14) in Frankfurt am Main, Germany.

### Recruitment

Participants will be recruited at different places in Germany. This is why dental and medical practices have been chosen to ask patients being there for medical check-ups. Furthermore, contacting companies directly via mail and telephone and using notice boards at universities are methods for recruitment. Participation is voluntary.

### Measurement systems

#### Three dimensional back scan

**Fig. 1:**
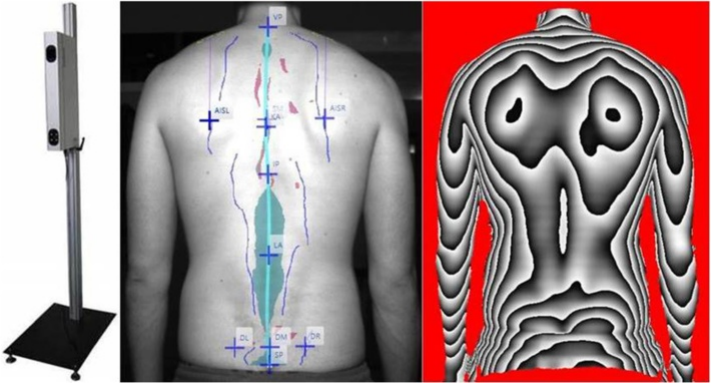
Back scanner MiniRot Combi (ABW GmbH, Frickenhausen/Germany) (left picture), marker placement on the bare back (central picture) and three-dimensional phase picture of the back (right picture)

The 3D back measurement device “MiniRot Kombi” (ABW GmbH, Germany) is able to record changes in the upper back posture while standing using the videorasterstereography (Fig. 1). The sampling frequency is 50 Hz with a spatial resolution of 1/100 mm.

An LCD camera captures this pattern from a defined angle.^1^ Therefore, the exposure of the back surface can be demonstrated as a phase picture in the software program (Fig. 1). For this phase picture all participants are marked with six markers on the skin which are defined anatomically (Fig. 1).

The measurement system needs approximately 2 s for the admission and data production of the six surface markers including the calculation and representation of the three-dimensional coordinates in a phase picture. During one sequence 15 photos are shot.

The measurement error is specified by the manufacturer with <1 mm. In reliability measurements the reproducibility is about <0.5 mm. This results from the fact that the calculations of the upper body posture are directly made by the marker on the object. This decreases artifacts that might be caused due to different placement of the patient in front of the scanner.

### Force measuring platform

Postural control will be detected using a force platform (GP MultiSens, GeBioM, Münster, Germany) with an array of 2304 pressure sensors on a 1.444 cm^2^ surface. The sensor is 8 mm^2^ with a high sensor resolution due to two sensors per square millimeter. The sensors are arranged in a 48 row and 48 line matrix with a sampling frequency of 200 Hz. Therefore, sensor density is 0,64 per cm^2^. After amplification by a high ohm-resistance multiplier, the signals will be analyzed with the software “GP Fussdruck” (GeBioM, Münster, Germany). The results will be displayed as color-coded mesh.

### Evaluation criteria

#### Upper body posture

**Fig. 2:**
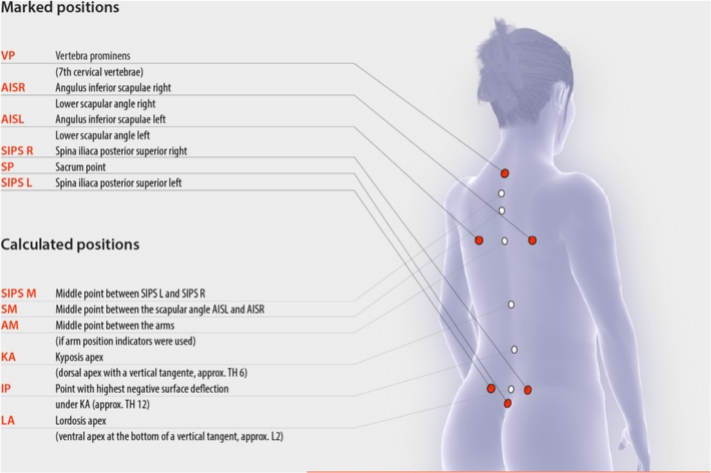
Marked and calculated positions of the back scan

The three-dimensional phase picture of the back will be divided into three components: spine (marker on C7 and L3), shoulder (marker at the highest place of the scapula) and pelvis (marker on left and right Spina iliaca posterior superior [SIPS]). All evaluation parameters are listed in Table 1. The illustration of the marker setup is shown in Fig. 2.

**Table 1:** Detailed list and explanation of all back scan parameters

**Spine parameter**	
Trunk length D (mm)	Spatial distance between the markers VP and DM
Trunk length S (mm)	Spatial distance between the markers VP and SP
Sagittal trunk decline (°)	Inclination of the trunk length D marked line from the perpendicular to the sagittal plane. Tilt anteriorly (negative values) = possible lordosis Tilt dorsally (positive values) = possible kyphosis
Frontal trunk decline (°)	Inclination of the trunk length D marked line from the perpendicular to the frontal plane. Tilt anteriorly (negative values) = possible lordosis Tilt dorsally (positive values) = possible kyphosis
Axis decline (°)	*Deviation of the line of the area marked by the trunk length D line* of the 90 ° rotated distance DL-DR →decline between upper body and pelvis
Thoracic bending angle (°)	Deviation of the distance VP - KA from the perpendicular
Lumbar bending angle (°)	Deviation of the distance KA - LA from the perpendicular
Standard deviation lateral deviation (mm)	Root mean squared deviation of the median line of the distance VP - DM
Maximal lateral deviation (mm)	Maximum deviation of the median line of the distance VP - DM Negative values = deviation to the left Positive values = deviation to the right
Standard deviation rotation (°)	Root mean square deviation of surface rotation of the median line (torsion of the spinous processes of the spine)
Maximal rotation (°)	Maximum positive or negative surface rotation on the median line
Kyphosis angle (°)	In the sagittal plane measured angle between the upper inflection point of the spine at the thoracolumbar and VP inflection point IP; point of greatest negative surface decline
Lordosis angle (°)	Angle between the inflection point at DM and the thoracolumbar inflection point IP
**Pelvis parameter**	
Pelvis distance (mm)	Spatial distance between SIPS L and SIPS R.
Pelvis height (°) and (mm)	Decline of the connecting line between SIPS L and SIPS R to the horizontal in the frontal plane in degrees and millimeter
Pelvis torsion (°)	Angle between the surface normal on the two dimples SIPS L and SIPS R Negative differential angle = Normal at point SIPS L is stronger upward as at point SIPS R Positive difference angle = Normal at point SIPS L is stronger downward as at point SIPS R.
Pelvis rotation (°)	Rotation of the distance SIPS L – SIPS R in the transversal plane
**Shoulder parameter**	
Scapular distance (mm)	Distance between the left (AISL) and the lower right scapular angle (AISR).
Scapular height (°)	Height difference between the points AISL and AISR Positive value = AISR higher than AISL Negative value = AISR deeper than AISL
Scapular rotation (°)	Rotation of the distance DL-DR in the transversal plane
Scapular angle left (°)/Scapula angle right (°)	Best fit straight line on the shoulders to the horizontal. The center point of the regression line is set vertically above AISL / AISR. The greater the angle, the more caudally located the shoulder.

### Postural control

To assess postural control, balance (% of weight distribution) is determined. Average balance is therefore the average weight distribution during the measurement period for each foot. Same will be performed for front and back foot as well as for the left and right foot.

Furthermore, the body sway area of frontal and sagittal excursion (mm) will be determined.

### Measurement protocol

All measurements will be performed in dimmed, quite rooms with comfortable room temperature.

Participants will be asked to stand barefoot with upper body undressed in habitual posture. Heels will be parallel to back scanner (distance: approximately 135 cm). Arms will hang down loosely with the view fixed at a point on the opposite wall on eye level. One measurement lasts for 5 s and will be repeated five times with short periods of rest in between.

### Statistical data analysis

#### Sample size

The aim of the study is the determination of ranges of tolerance and 95 %-confidence intervals for upper body posture and postural control depending on age and gender. Using a sample size of *N* = 100 per decade and gender the precision of the corresponding 95 %-tolerance interval will be 3.7 % with a 95 % probability [32].

The determination of general ranges of tolerance intervals and confidence intervals for upper body posture and postural control parameter will be based on models adjusting for age and gender and will result in a higher precision:

Aiming at a sample size of *N* = 1000, we can assure that that the coverage of the tolerance interval is no more than 96.8 % with 95 % confidence [32].

### Data analysis

Data will be tested for normal distribution using Kolmogorov-Smirnoff-Lilliefors-Test. Depending on the distribution, 95 %-confidence intervals for the mean or (in case of non-normality) for the median and normal or non-parametric 95 %-tolerance intervals with 99 % confidence (this is the confidence with which a tolerance interval based on *sample* mean and *sample* standard deviation actually includes the specified proportion of the population) will be constructed. The analysis comprises the evaluation parameters of the back scan and the force plate described above.

In addition the correlation between the upper body and the postural control parameters will be analyzed by Pearson- or Spearman-Rank-correlation. For this explorative analysis we will provide single test *p* –values and a correlation matrix heat map.

Furthermore, all parameters will be tested for specific group differences using the Two-sample-T-test or the Mann-Whitney-U-test. Hereby, *p*-values will be adjusted via Bonferroni-Holm.

All tests will be two-sided with a significance level of 5 %.

Statistical analyses will be performed using BiAS 11.0 (Epsilon Verlag, Norderstedt/Germany). The correlation matrix heat map will be constructed using R (R Core Team (2013). R Foundation for Statistical Computing, Vienna, Austria).

## Discussion

Up to now, no standard values and confidence intervals of upper body posture and postural control have been reported, in which both parameters were evaluated synchronously, for different age, gender and occupational groups. Concerning posture and postural control, there have been many trials for classification in the past.

One of the earliest attempts to define normal values was conducted by Staffel in 1889 [33]. Based on the attempt to describe a perfect posture, German orthopaedics have established different procedures, e.g. postural index by Fröhner [34, 35] or Matthiass-Test [36, 37]. Flügel et al. [38] have measured the population of the German Democratic Republic to obtain standard values. The aim was to assess growth and development, partly also the health and resilience and controlling the success of therapeutic interventions. Another possibility for diagnostics is to measure the distance from the plump line to a wall which the person is leaning on. Every deviation from this functional equilibrium leads to a shift and therefore increases stress on active and passive structures [39–41].

The standard values will help decision making for therapeutic or clinic interventions as well as in the evaluation of therapeutic interventions.

### Limitations

Having the expected difficulties of these planned investigations in mind, the Hawthorne effect should be considered as potential factor which may have an impact on the results. It is a possible explanation for the distortion of results in non-blind intervention studies since it involves behavioral changes due to awareness of being observed. Here, the active compliance of the test person and the presumed wishes of researchers are combined [42]. In contrast, a distortion of the results by the investigator is not likely to have an influence on this analysis of the restricted results due to pure numbers and defined evaluation criteria [43].

Videorasterstereography has been shown to be a valuable tool to determine back geometry in several studies. In comparison with other methods Asamoah et al. [44] found it to be effective to diagnose scoliosis and certain deformations. Values for sensitivity and specificity were 98 and 84 %. Other authors reported good correlations of angle measurements using videorasterstereography and X-ray [45] (*r* >.8 to .93). Restrictions were found with overweight people and people with extremely asymmetric back musculature. Asamoah et al. [44] postulated obesity becoming a problem for the measurements. Marker points have also been shown to be reproducible [46], but differences for parameters have been reported: angel measures are more prone to errors than distances. Drerup and Hierholzer [47] reported localization errors in a range of approximately 1 mm concerning Spina iliaca anterior superior. Fixation of markers is more complicated if participants are hairy and/or obese. Manual marker placement after the measurement may be helpful in this case.

## Conclusions

This project aims at improving classifications in adaptations of upper body posture and postural control. Measured standard values have not been determined before to this extent. Therefore, interventional effects may become better quantifiable and justiciable.

## Abbreviations

BMI, Body Mass Index; CNS, central nervous system; COP, centre of pressure; SIPS, Spina iliaca posterior superior

## Footnotes

1 This angle is determined by the permanent installation of the camera and projector in the unit.
